# Laser Microdrilling of Slate Tiles

**DOI:** 10.3390/ma12030398

**Published:** 2019-01-28

**Authors:** Joaquín Penide, Antonio Riveiro, Ramón Soto, Mohamed Boutinguiza, Felipe Arias-Gonzalez, Jesús del Val, Rafael Comesaña, Fernando Lusquiños, Félix Quintero, Juan Pou

**Affiliations:** 1 Department of Applied Physics, University of Vigo, EEI, Lagoas-Marcosende 9, 36310 Vigo, Spain; jpenide@uvigo.es (J.Pe.); rfsoto@uvigo.es (R.S.); mohamed@uvigo.es (M.B.); felipeag@uvigo.es (F.A.-G.); jesusdv@uvigo.es (J.d.V.); flusqui@uvigo.es (F.L.); fquintero@uvigo.es (F.Q.); jpou@uvigo.es (J.Po.); 2 Department of Materials Engineering, Applied Mechanics and Construction, University of Vigo, EEI, Lagoas-Marcosende, 36310 Vigo, Spain; racomesana@uvigo.es

**Keywords:** CO_2_ laser, drilling, slate tiles

## Abstract

Slate is a natural rock usually used in roofs, façades, and for tiling. In spite of this broad use, the production process of slate tiles requires substantial improvements. An important quantity of slate from the quarry is wasted during the manufacturing of the final product. Furthermore, processes are not automatized and the production lead times can be considerably shortened. Therefore, new processing methods to increase productivity, reduce costs and to provide added value to the final slate product are required. Drilling is an important part of these manufacturing processes. Conventional drilling processes usually cause the breaking of the slate tiles; then, even a higher quantity of material is wasted. To overcome these problems, lasers emerge as a feasible tool to produce holes in this material, since mechanical stresses are not induced on the workpiece. In this work, we have studied the CO_2_ laser microdrilling of slate tiles. We used a Design of Experiments (DOE) methodology to determine the influence of the laser processing parameters on the hole quality. This work demonstrates the capability of a CO_2_ laser to produce holes in slate with less than 100 microns in diameter, avoiding any fracture, and with a processing time of less than 50 ms per hole. Finally, this process demonstrates the viability of the production of high-density micron-sized holes in a slate tile for water draining purposes.

## 1. Introduction

Slate is a natural rock, typically of a dark grey color. It is widely used in the building industry, partly because of its attractive rustic appearance, high wear resistance, and good frost resistance [1]. Particularly, more than 1 billion tons per year are manufactured in the world, and this market reaches, for example more than 500 million dollars per year in Spain alone [2]. Typical uses of slate are for outside of buildings, in roofs or façades [1], as well as for inside, for tiling bathrooms or fireplaces. This material is even used in dinnerware: dishes made of slate have an interesting and different appearance, which is really appreciated in the restaurant and catering sectors.

The manufacturing of slate tiles is performed in several steps [3]. First, large slate blocks are cut with diamond wire sawing machines in the quarry. Then, these are converted into plates no wider than 30–35 cm due to their anisotropy in strength [4]. Finally, they are split again into 3–5 mm thick tiles. The final parts are produced under the required dimensions using a die. However, in spite of the broad use of slate, these manufacturing processes have remained unaltered for many years, and they are highly inefficient. It is estimated that more than 90% of the material is wasted during the transformation stages from the quarry up to the final slate tile. Furthermore, conventional manufacturing processes provide a low added value to the final slate parts. An example of this is the slate drilling itself. In this case, holes are used, in general, for fixation purposes as slate tiles are traditionally fixed to the roof by nails. However, traditional drilling methods induce mechanical forces on the tiles, and some of them break accidentally. Therefore, hooks are more commonly used for fixation purposes to avoid this additional waste of material [5].

An interesting and new use of holes in slate tiles, providing also a high added value, is the drilling of microholes for air ventilation, water draining, or filtration applications. For example, the United States Environmental Protection Agency (EPA) suggested that the water employed in showers can be reused either for toilet or for irrigation purposes [6]. Therefore, water could be pre-filtered (or separated from big particles) by making small holes in the slate. These applications require micron-sized holes. In general, pre-filters used to separate sand (e.g., in rainwater systems) have a pore size in the range of 50–100 µm. However, conventional drilling processes can damage the tiles as mentioned earlier, especially when a high-density of microholes are required. Mechanical stresses produced by conventional drilling tools can break the slate tiles either in the moment of nailing, or in the worst cases some days later after finishing the process [5].

Laser drilling is a feasible alternative to produce a high-density array of microholes in slate tiles for filtration applications. This technique does not induce any mechanical stress in the workpiece, avoiding the formation of cracks in the final part. Moreover, it is also a reliable and easily automatable process, which is able to produce reproducible holes in very short times. Laser-drilled holes can be considerably smaller as compared to those produced with conventional drilling tools [7]. Hole diameters from hundreds of nanometers up to several tens of millimeters can be easily fabricated using different laser drilling techniques [8]. When the hole diameter is less than 1 mm (but larger than the sub-μm range), then this process is termed as laser microdrilling. This technique has been tested in many different materials (metals, polymers, ceramics, or composites), and for many industrial applications [8], such as in in fuel injector nozzles, microfluidic devices, and in electronic packaging materials. Nasrollahi et al. developed a two-side laser processing method to drill 250-µm thick silicon nitride (Si_3_N_4_) substrates [9]. Holes with less than 60 µm in diameter and taper angle less than 10°, suitable for electronic applications, were produced. Tagliaferri et al. demonstrated the possibility to drill 0.4 mm thick NIMONIC® 263 sheets, using a trepanning technique as an alternative to produce holes for effusion cooling systems used in aero-engines [10]. Maclean et al. studied the production of microholes in Si semiconductor wafers using millisecond laser pulses [11]. Above a threshold irradiance, cracks are almost avoided. Ito et al. investigated the damage mechanisms leading to the formation of cracks during femtosecond laser microdrilling of glass [12]. These mechanisms are related to thermal stresses in the hole entrance and to stress waves in the hole walls and exit. Another approach to produce microholes is the utilization of ultrashort laser pulses. Narollahi et al. studied the influence of the laser fluence and the focal length on the morphology of the microholes during the laser percussion microdilling of 250 μm silicon nitride wafers [13]. Hole quality increased with the laser fluence and the reduction of the focal distance. Different works also demonstrated the possibility of producing a high-density array of microholes suitable for different applications. For example, Wu et al. used a Ti:sapphire femtosecond laser system to produce an array of microholes (ranging from 5 to 42 μm) in a titanium foil, suitable for oil–water and oil–oil separation [14]. Similarly, Zhao and Wang demonstrated the possibility to drill a high density of microholes through flexible printed circuits made of several layers of copper and polyimide [15]. Therefore, laser microdrilling could be a potential approach to produce a high-density array of microholes in slate for water draining and filtration purposes.

Laser drilling, and in particular microdrilling, is a process depending on many processing parameters. This dependence is rather complex, and largely relies on the material under investigation. Statistically planned experiments are suitable to study this kind of complex processes. They have been used in the study of many different laser processes [16], such as in laser-assisted coating [17], laser cutting [18,19], laser bending [20], but also in laser drilling [21,22,23]. Bharatish et al. [24] used this methodology to determine the influence of the processing parameters in the final quality (i.e., circularity and heat affected zone) of holes produced on alumina. On the other hand, Nagesh et al. [25] used a design-of-experiments (DOE) methodology to study quality aspects of the process such as the heat affected zone (HAZ) and taper angle in holes performed on carbon composites. Response surface method (RSM) statistical approach was used by Biswas et al. to optimize the processing parameters during the Nd:YAG laser microdrilling of alumina–aluminium interpenetrating phase composites [26].

In the case of slate, an initial study on the CO_2_ laser drilling process was done by Boutinguiza et al. [27]. Potential production of sub-millimeter holes in slate tiles was confirmed; however, the micron-sized dimensions required for water draining or filtration applications were not obtained. Similar results were obtained by Lusquiños et al. using a Nd:YAG laser [28]. Holes larger than 400 µm in diameter were produced but with lower quality. Therefore, the aim of this work is the in-depth study of the whole laser drilling process of slate tiles to produce a high-density array of micron-sized holes. Statistically planned experiments were performed to determine the influence of the different processing parameters on the hole diameter. Finally, the optimum processing parameters to produce holes with about 100 µm in diameter were determined. This hole diameter was considered suitable because it is in the same range than the pore size of pre-filters used for water filtration purposes. The applicability of this procedure for filtration applications was also demonstrated.

## 2. Materials and Methods

In this study, we used 3.5 to 5 mm thick slate tiles (Valdeorras variety) with dimensions of 300 mm × 200 mm. This natural rock is a foliated metamorphic rock, also called layered rock, because it was formed layer by layer; it is thus easy to split it into thin plates. Table 1 summarizes the mineral content (in wt.%) for the slate variety used during these experiments. The main constituent of slate is quartz. Boutinguiza et al. measured the thermophysical properties of slate in the parallel (radial, i.e., along one layer) and perpendicular (axial, i.e., between layers) directions to the layers forming a slate tile (Valdeorras variety) [29]. The heat conduction is higher along the layers than between the layers, as seen in Table 2.

Laser drilling experiments were performed using a 3.5 kW CO_2_ slab laser (ROFIN DC-035, ROFIN-SINAR Laser GmbH, Hamburg, Germany), the laser mode being TEM_00_. A CO_2_ laser was selected due to the high laser absorptivity of silica and high-silica content materials (as in the present case) for this laser wavelength (λ = 10.6 μm). Although it is possible to use 1 μm laser sources (e.g., Nd:YAG or fiber lasers), the quality of the processed areas with this wavelength is lower, as demonstrated in the literature (see Refs. [27,28,30]). On the other hand, CO_2_ slab lasers have an excellent beam quality (K ≥ 0.9) and focusability, both suitable for laser microdrilling.

The laser beam was focused on a spot of 95 μm in diameter on the surface of the workpiece using a 127 mm focal length lens. Air was used as assist gas in all the experiments.

We used a design of experiments (DOE) methodology to determine the influence of each processing parameter on the hole entrance and exit diameters. A L8 orthogonal array (Taguchi design) was used to fix the combination of levels of each factor in all the trials. Eight factors were chosen at three levels (see Table 3). The studied factors were: the average laser power (P), pulse frequency (F), number of pulses (N), duty cycle (factorial amount of time the laser is “on” during the pulse length) (C), air pressure (G); stand-off distance (distance between the laser head and the surface of the slate) (S), nozzle diameter (B), and focus position (FP). Three replicates were done for each combination of parameters. The results of this analysis were evaluated in terms of the influence of the processing parameters on: (1) the hole entrance diameter laser entry side and (2) the hole exit diameter (laser exit side).

Next, we studied the interdependence of some of the processing parameters. In this case, three factors at three levels were analyzed: pulse frequency (F), focus position (D), and duty cycle (C). Table 4 summarizes the values of these factors. In this case, we made 15 trials with two replicates. As in the previous study, the results were evaluated in terms of the hole entrance and exit diameters.

Hole diameters were determined using an optical microscope (Nikon, SZM-10, Tokyo, Japan) coupled to a micrometric XY table. Three diameters were measured for each hole, and an average value was extracted. Furthermore, the circularity of selected drilled holes was also evaluated at the hole entrance (C_ent_) and exit (C_exit_). This was measured as the ratio of the minimum to the maximum Feret’s diameter at the hole entrance and exit [26].

The morphology of the holes was evaluated using scanning electron microscopy (SEM Philips XL30, North Billerica, MA, USA). X-ray diffraction (X’Pert PRO X-Ray Diffractometer, PANalytical, Almelo, Netherlands) analyses of untreated and laser-treated areas in slate were performed to analyze the difference in microstructure after the laser processing.

## 3. Results and Discussion

### 3.1. Influence of Processing Parameters

**Figure 1:**
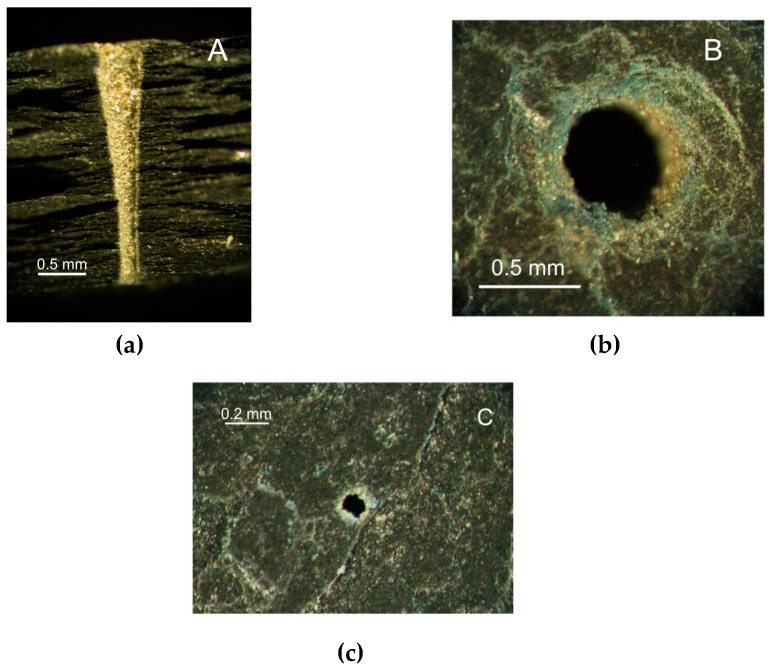
Optical micrographs of the (**a**) cross-section, (**b**) entrance side, and (**c**) exit side of a hole produced with the CO_2_ laser (Processing parameters: P = 300 W, S = 3 mm, F = 2000 Hz, N = 45, C = 50%, FP = −1 mm, B = 3 mm, G = 6 bar).

Typical holes produced by laser drilling are either cylindrical or conical, depending on the combination of processing factors (see Figure 1). Natural layers of slate can be easily distinguished in the cross section (see Figure 1a). As seen, the entrance diameter is larger than the exit (see Figure 1b,c); however, this difference depends on the processing conditions. Holes are not perfectly circular. The hole circularity at entrance is C_ent_ = 0.94, while for the exit is C_int_ = 0.77. The reduction in circularity at the exit is probably a consequence of the degradation of the beam quality due to the interaction of the laser beam with the vapor/plasma plume (containing particles) during the drilling process.

**Figure 2:**
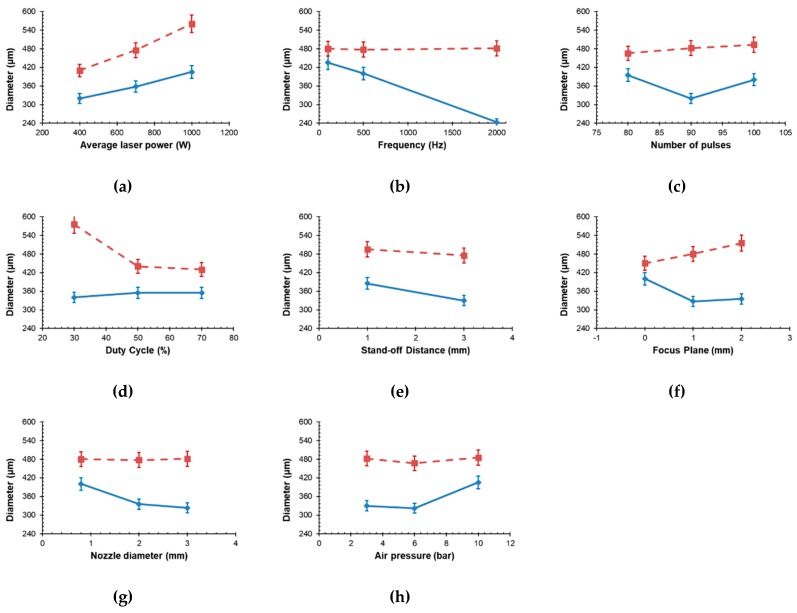
Main effects plots showing the influence of the processing parameters: (**a**) laser power; (**b**) frequency; (**c**) number of pulses; (**d**) duty cycle; (**e**) stand-off distance; (**f**) focus position; (**g**) nozzle diameter; (**h**) gas pressure on the entrance (dotted line) and exit (continuous line) diameters.

Figure 2 shows the main effects plot for each factor on the entrance and exit diameters. As seen, the most influential factors on the entrance diameter are laser power, duty cycle and focus position. In the case of laser power (Figure 2a), the size of the entrance diameter is linearly dependent on the laser, as the laser energy determine the extension of the affected area. On the other hand, duty cycles lower than 50% produce large entrance diameters, while for values higher than 50%, the size is almost constant (Figure 2d). These results can be related to the variation of the peak power with the duty cycle. Low duty cycles give high peak powers. Under these conditions, the removal of material is more effective for high peak powers, probably due to a higher evaporation regime. A similar effect is obtained with the position of the focal spot position (or in short focus position). The entrance diameter is smaller when the laser beam is focused just on the surface of the slate tiles (Figure 2f). In this case, the power (or energy) density on the surface of the workpiece is maximum, and it is concentrated in a reduced area (spot diameter around 110 µm); then, the evaporation of material is more intense. The rest of processing factors seem to have a more limited influence on the entrance diameter.

Regarding the exit diameter, this response is mainly affected by the laser power and frequency. The influence of the other parameters is more restricted. Laser power has an analogous influence to that in the case of the entrance diameter (Figure 2a). The lower the laser power, the smaller the exit diameter. On the contrary, the pulse frequency has the opposite effect: the higher the frequency, the smaller the exit diameter (Figure 2b). Higher frequencies lead to higher amounts of ablated material rather than melted, and it seems to be beneficial to the production of smaller holes. Regarding the stand-off distance, lower distances lead to larger holes (Figure 2e). Focus position should be fixed under the surface of slate if we aim to minimize the exit diameter (Figure 2f); in this way, the focal spot will be closer to the exit side of the hole. The nozzle diameter determines the speed of the assist gas, and the removal capabilities of molten and evaporated material (Figure 2g). The smaller the nozzle diameter, the larger the exit diameter, because more melted and evaporated material by the action of the laser beam is extracted from the interaction zone. Smaller nozzle diameters remove the molten material more efficiently because: (1) the occlusion of the jet is lower for small jets, (2) the jet velocity (and the removal capacity) is higher [18]. Gas pressure has a similar influence. For high enough pressures, the removal of molten material is more efficient and the exit diameter increases.

**Figure 3:**
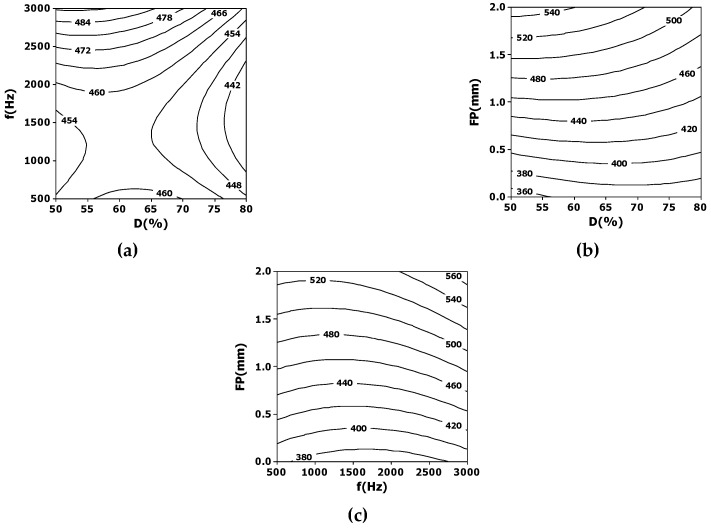
Contour plots showing the interdependence of some laser processing parameters, namely: (**a**) duty cycle and frequency; (**b**) duty cycle and focus position; (**c**) focus position and frequency, on the average entrance diameter (in microns).

**Figure 4:**
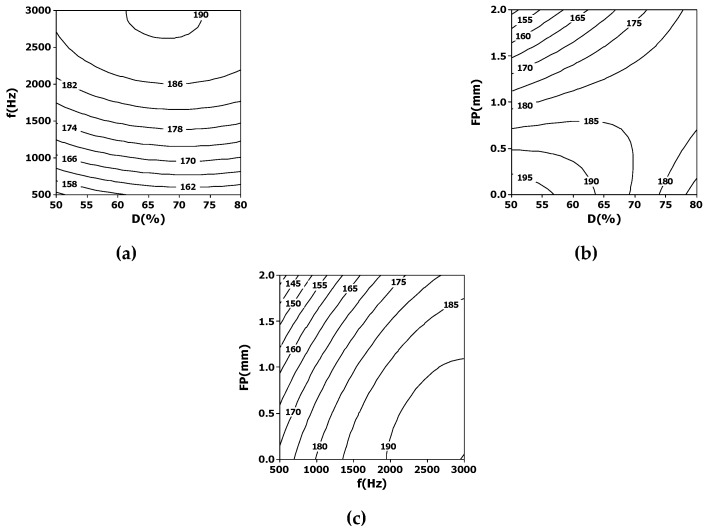
Contour plots showing the interdependence of some laser processing parameters, namely: (**a**) duty cycle and frequency; (**b**) duty cycle and focus position; (**c**) focus position and frequency, on the average exit diameter (in microns).

Figure 3 and Figure 4 summarize the results relative to the simultaneous influence of duty cycle, pulse frequency, and focus position. Figure 3 shows the contour plots relative to the influence on the entrance diameter. As seen in Figure 3a, the hole diameter is lower for higher values of duty cycle and medium values of pulse frequency (around 1000–2000 Hz); however, the difference in the final diameter is not highly significant. On the other hand, there is a slight interaction of the duty cycle for higher values of focal spot position (Figure 3b); this interaction is almost absent for low values of focal spot position (namely, when laser beam is focused on the entrance surface). Figure 3c shows a similar behavior for the interaction between focus and frequency. In this case, the best way to produce the smallest entrance diameter is by focusing the laser beam on the entrance surface of the slate with an almost non-dependent duty cycle or pulse frequency.

Regarding the exit diameter, results are shown in Figure 4. It seems that lower frequencies are better to produce smaller holes in this case (Figure 4a). The duty cycle and focus position have an interesting interaction (Figure 4b). For low values of duty cycle, small exit diameters are obtained if the laser beam is focused deeper on the slate tile; on the contrary, the trend is the opposite for high duty cycles. The best choice for producing small exit diameters is 50% of duty cycle and focusing laser beam as close as possible to the exit surface. Figure 4c shows a similar trend for the interaction focus position and pulse frequency. The variation of the exit diameter with the focus position is more pronounced for low pulse frequencies.

### 3.2. Drilling under Optimum Conditions

After analyzing the influence of the processing parameters on the hole diameter, the optimum processing conditions to produce micron-sized holes were determined. The analysis of the previous results suggest that the laser power should be as low as possible. Pulse frequency does not have any influence on the entrance diameter, but it is significant on the exit diameter. Small holes were produced using a pulse frequency around 2000 Hz. The number of pulses seems to be not relevant on the diameters. This parameter only determines the processing speed. For 3.5–5 mm thick slate tiles, the required number of pulses is in the range of 50–90 pulses. The optimum value for the duty cycle seems to be higher than or equal to 50%; lower values for the duty cycle increase the hole diameter. Regarding the stand-off distance, the better results were obtained for a distance of 3 mm. No clear value or range is observed for the focal spot position; in this case, it is required to find a compromise between entrance and exit diameter because the effect is opposite. In this case, the focal spot position was placed 1 mm under the surface of slate. Regarding the parameters related to the assist gas, a nozzle diameter around 3 mm and an assist gas pressure of around 6 bar led to the better results. Table 5 shows a summary of the optimized processing parameters required to produce holes with an exit diameter of 100 ± 20 μm (suitable for water draining and filtration purposes).

**Figure 5:**
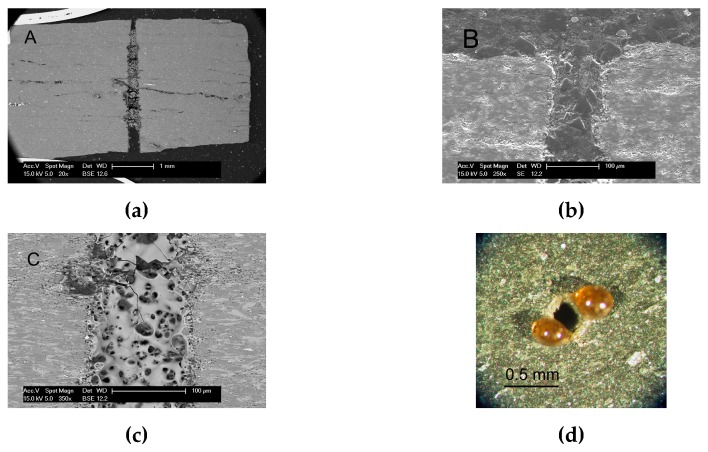
SEM images of the cross-section of a hole produced under optimized processing parameters: (**a**) overall view; (**b**) detail of the exit surface; (**c**) detail of the resolidified material; (**d**) Optical micrograph showing some resolidified dross adhered to the exit side of a hole.

**Figure 6:**
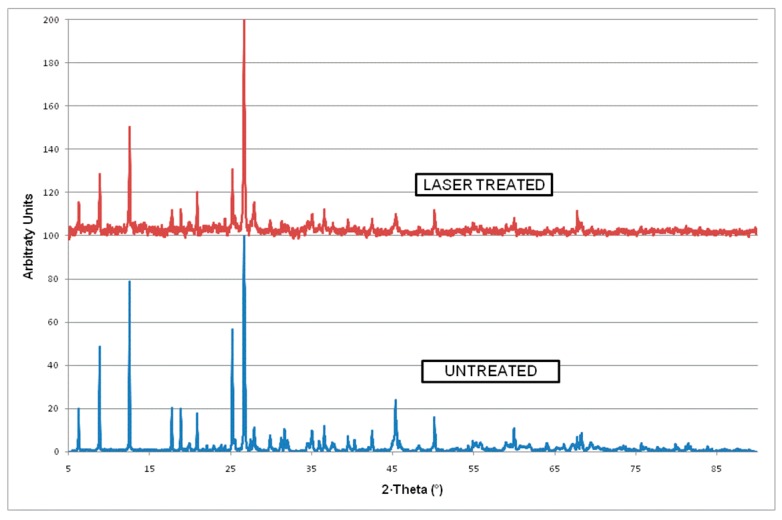
X-Ray Diffraction analysis of the laser-treated (resolidified material) and the untreated (or base) material.

Using these optimized processing parameters, micron-sized holes were produced on 3.5–5 mm thick slate tiles (see Figure 5a). The hole shows a conical shape with an entrance diameter of 350 µm and 100 µm on the exit surface (Figure 5b), approximately. The hole circularity at entrance is C_ent_ = 0.95, while at the exit is C_int_ = 0.81. Some resolidified material is observed covering the wall of the holes (see Figure 5c). This layer has a typical thickness around 20 μm for the holes made with the parameters summarized in Table 5. This resolidified material (with a brown bright color) is occasionally adhered to the exit side of the hole in form of dross (Figure 5d). This molten material usually remains stuck with a spherical shape. Its adhesion to the surface of the slate is weak, and it can be easily removed without any specific tool. X-Ray diffraction analyses were performed to compare the structural differences between this resolidified material (i.e., laser-treated material) and the base material (untreated material). Figure 6 summarizes this comparison. The peak intensities in the laser-treated slate (i.e., in the resolidified material) is in general smaller than for the base material. Therefore, a general amorphization of the slate minerals is produced under the action of the laser radiation.

**Figure 7:**
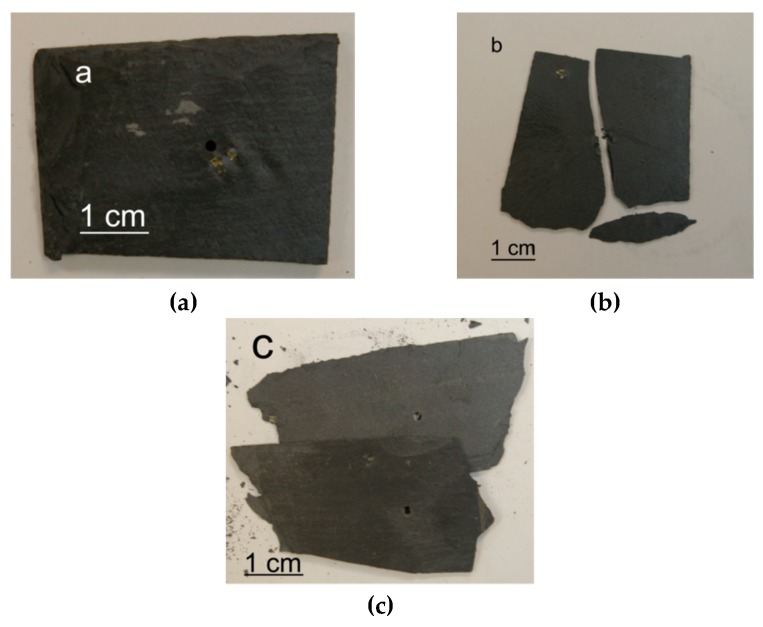
(**a**) Hole performed in a 5 mm thick slate using a conventional drilling tool (1.5 mm in diameter). Examples of typical breaking modes after conventional drilling processes: (**b**) cracking and (**c**) foliating.

**Figure 8:**
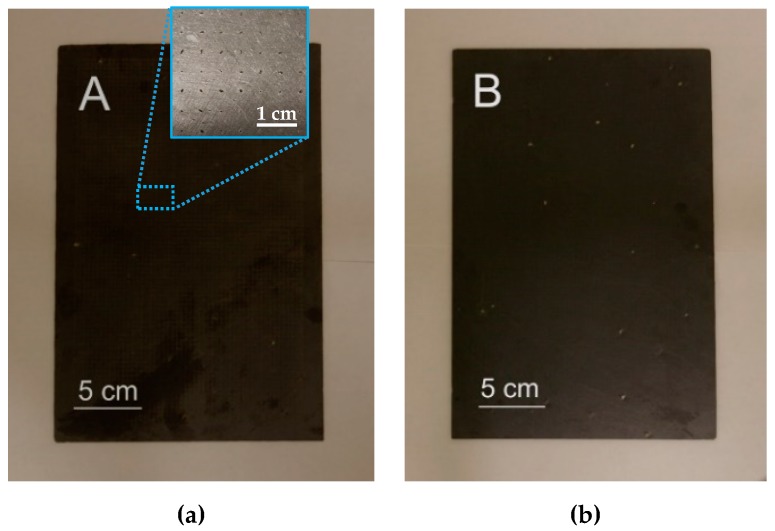
Images of the (**a**) entrance surface and (**b**) exit surface of a slate tile where 4082 holes were laser drilled under optimized processing parameters.

**Figure 9:**
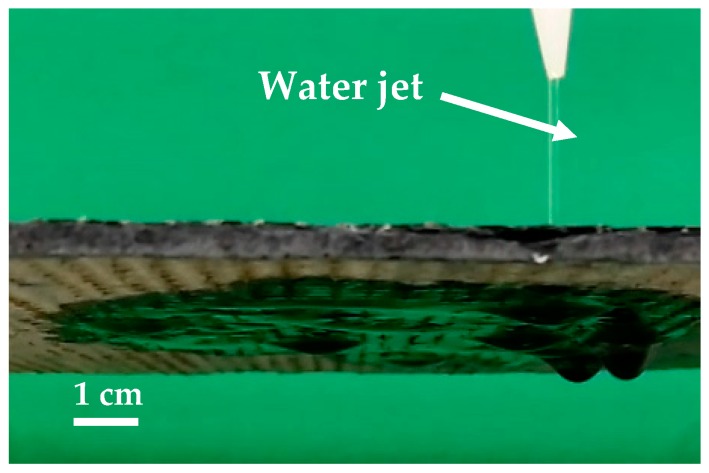
Image of a laser-drilled slate tile demonstrating the capability to act a water filter. A water jet is supplied to the upper face of the slate tile; water easily passes through the holes up to the lower face.

This work thus demonstrates the possibility to produce 100 ± 20 µm holes in 3.5–5 mm thick slate tiles using a CO_2_ laser. Under these optimized processing conditions, the processing time to produce a hole is less than 50 ms. This processing time is shorter than in conventional drilling processes [7]. In Figure 7a, a conventional drill, 1.5 mm in diameter, is depicted. As observed, the mechanical action of the drilling tool can produce the breaking of the tile in two main ways [5]: cracking (Figure 7b) or foliating (Figure 7c). This problem would be aggravated if multiple holes were produced in the same workpiece. On the contrary, Figure 8 shows a slate tile, where more than 4000 holes were laser drilled. These are difficult to see with the naked eye due to their reduced dimensions, especially in the exit side. However, the generation of such an amount of holes in slate tiles is very interesting for draining or water filtration applications (e.g., draining water in a fountain). As depicted in Figure 9, laser drilling a high density of micron-sized holes makes the processed slate tiles permeable to water. Thus, water supplied on one side easily passes through the slate tiles (see Video S1). In this way, water can be easily drained or even separated from particles larger than 100 µm.

## 4. Conclusions

In this work, the ability of a CO_2_–slab laser to produce 100 μm holes in less than 50 ms per hole on 3.5–5 mm thick slate tiles was demonstrated. The influence of the processing parameters on the hole diameter was determined using statistically planned experiments. This result allows the production of a high-density of such micron-sized holes in a reduced time, and with the minimum modification of the slate tile in terms of final appearance and mechanical properties. Therefore, we can conclude that CO_2_ lasers are a reliable tool for microdrilling slate. Holes of any desired diameter can be produced, with slate tiles never breaking during this process, and can be quickly and easily automatable.

## Figures and Tables

**Table 1:** Mineral content (wt.%) for slate tiles (Valdeorras variety) estimated using a semiquantitative X-ray diffraction (XRD) methodology.

Mineral	Content (wt.%)
Silica (Quartz)	55.9
Clinochlore	19.1
Titanium oxide	2.3
Albite (Feldspar)	10.6
Illite (Mica)	5.1
Muscovite (Mica)	7.0

**Table 2:** Thermophysical properties along the parallel (radial) and perpendicular (axial) direction to the layers forming a slate tile (Valdeorras variety) [29].

	Radial Direction	Axial Direction
Density (kg/m^3^)	(2.7 ± 0.1) × 10^3^	
Heat capacity (kJ/kgK) @ 293–773 K	0.96 ± 0.04	
Thermal conductivity (W/mK)	5.2 ± 0.80	2.00 ± 0.2
Thermal diffusivity (mm^2^/s)	2.02 ± 0.15	0.75 ± 0.01

**Table 3:** Factors and levels corresponding to the L8 orthogonal array (a focus position of 0 mm corresponds with the focal spot on the surface of the slate tile; negative values refer to positions underneath the surface).

Factor	Level		
(S) Stand-off (mm)	1	3	-
(P) Average laser power (W)	400	700	1000
(F) Frequency (Hz)	100	500	2000
(N) Number of pulses	80	90	100
(C) Duty cycle (%)	30	50	70
(FP) Focus position (mm)	0	−1	−2
(B) Nozzle diameter (mm)	0.8	2	3
(G) Air pressure (bar)	3	6	10

**Table 4:** Factors and levels used in the interdependence study (a focus position of 0 mm corresponds with the focal spot on the surface of the slate tile; negative values refer to positions underneath the surface).

Factor	Level		
(F) Frequency (Hz)	500	1750	3000
(FP) Focus position (mm)	0	−1	−2
(C) Duty cycle (%)	50	65	80

**Table 5:** Optimized values for the processing parameters.

**Laser Power (P)**	**Stand-Off (S)**	**Frequency (F)**	**Number of Pulses (N)**
165 W	3 mm	2000 Hz	90
**Duty Cycle (C)**	**Focus Position (FP)**	**Nozzle Diameter (B)**	**Gas Pressure (G)**
50%	−1 mm	3 mm	6 bar

## Supplementary Materials

The following are available online at http://www.mdpi.com/1996-1944/12/3/398/s1, Video S1: Water filtration after laser microdrilling of slate tile.
